# Retraction: Tanshinone IIA alleviates lipopolysaccharide‐induced acute lung injury by downregulating TRPM7 and pro‐inflammatory factors

**DOI:** 10.1111/jcmm.18313

**Published:** 2024-04-23

## Abstract

Jie Li, Yan Zheng, Ming‐Xian Li, Chu‐Wei Yang, Yu‐Fei Liu, Tanshinone IIA alleviates lipopolysaccharide‐induced acute lung injury by downregulating TRPM7 and pro‐inflammatory factors. *Journal of Cellular and Molecular Medicine*, 22: 646–654. https://doi.org/10.1111/jcmm.13350

The above article, published online on 18 October 2017 in Wiley Online Library (wileyonlinelibrary.com), has been retracted by the journal Editor‐in‐Chief, Stefan Constantinescu, The Foundation for Cellular and Molecular Medicine, and John Wiley and Sons Ltd. The retraction has been agreed upon following an investigation into concerns raised by a third party, which revealed inappropriate duplications of image panels within the article (Figures 1 and 3A) as well as between this (Figure 1) and two other articles that were published previously in different scientific context. Thus, the editors consider the conclusions of this manuscript substantially compromised. The authors were informed about the concerns and the decision to retract but remained unresponsive.

Jie Li, Yan Zheng, Ming‐Xian Li, Chu‐Wei Yang, Yu‐Fei Liu, Tanshinone IIA alleviates lipopolysaccharide‐induced acute lung injury by downregulating TRPM7 and pro‐inflammatory factors. *Journal of Cellular and Molecular Medicine*, 22: 646–654. https://doi.org/10.1111/jcmm.13350

The above article, published online on 18 October 2017 in Wiley Online Library (wileyonlinelibrary.com), has been retracted by the journal Editor‐in‐Chief, Stefan Constantinescu, The Foundation for Cellular and Molecular Medicine, and John Wiley and Sons Ltd. The retraction has been agreed upon following an investigation into concerns raised by a third party, which revealed inappropriate duplications of image panels within the article (Figures 1 and 3A) as well as between this (Figure 1) and two other articles that were published previously in different scientific context. Thus, the editors consider the conclusions of this manuscript substantially compromised. The authors were informed about the concerns and the decision to retract but remained unresponsive.

